# The Co-operation of RUNX1 with LDB1, CDK9 and BRD4 Drives Transcription Factor Complex Relocation During Haematopoietic Specification

**DOI:** 10.1038/s41598-018-28506-7

**Published:** 2018-07-10

**Authors:** Jane Gilmour, Salam A. Assi, Laura Noailles, Monika Lichtinger, Nadine Obier, Constanze Bonifer

**Affiliations:** 1 Institute of Cancer and Genomic Sciences, College of Medical and Dental Sciences, University of Birmingham, Birmingham, B15 2TT UK; 2 Present Address: Centre for Clinical Research, University of Freiburg Medical School, Freiburg, Germany

## Abstract

Haematopoietic cells arise from endothelial cells within the dorsal aorta of the embryo via a process called the endothelial-haematopoietic transition (EHT). This process crucially depends on the transcription factor RUNX1 which rapidly activates the expression of genes essential for haematopoietic development. Using an inducible version of RUNX1 in a mouse embryonic stem cell differentiation model we showed that prior to the EHT, haematopoietic genes are primed by the binding of the transcription factor FLI1. Once expressed, RUNX1 relocates FLI1 towards its binding sites. However, the nature of the transcription factor assemblies recruited by RUNX1 to reshape the chromatin landscape and initiate mRNA synthesis are unclear. Here, we performed genome-wide analyses of RUNX1-dependent binding of factors associated with transcription elongation to address this question. We demonstrate that RUNX1 induction moves FLI1 from distal ETS/GATA sites to RUNX1/ETS sites and recruits the basal transcription factors CDK9, BRD4, the Mediator complex and the looping factor LDB1. Our study explains how the expression of a single transcription factor can drive rapid and replication independent transitions in cellular shape which are widely observed in development and disease.

## Introduction

Commitment to a specific cell fate requires a complex regulatory network of transcription factors acting in a stage-specific manner^1^. Significant progress has been made in identifying the transcription factor (TF) networks that are required to specify independent cell lineages during haematopoiesis. Wilson *et al*. identified a heptad of TFs essential for regulation of human HSPCs including GATA2, SCL/TAL1, RUNX1 and FLI1^2^. A smaller network of RUNX1, FLI1 and NF-E2 is responsible for terminal megakaryocyte differentiation, although other factors are necessary earlier in the differentiation process^3^.

The first definitive blood cell progenitors arise from specialised endothelial cells within the dorsal aorta which are termed the haemogenic endothelium (HE), and this process is known as the Endothelial to Haematopoietic Transition (EHT)^4–8^. RUNX1 plays an essential role in the EHT, since in its absence progenitor cells fail to emerge^9–11^. RUNX1 acts as both a repressor and activator of gene expression and both activities are essential for the EHT^10,12^. The importance of RUNX1 for the differentiation of multiple haematopoietic lineages suggests that the precise molecular mechanism governing the initiation of different transcriptional programs may derive from its many interaction partners.

Using an established model of Embryonic Stem Cell (ESC) differentiation we previously examined the role of RUNX1 in the onset of a haematopoietic transcriptional program. We employed an inducible RUNX1 (iRUNX1) ES cell line expressing an HA-tagged, doxycycline (Dox) inducible RUNX1 protein in a RUNX1−/− background^10,13^. In the absence of RUNX1 the HE is formed, but cells are unable to undergo the EHT and to form definitive haematopoietic precursors expressing the surface marker CD41. We showed that many haematopoietic genes required for the EHT are in a ‘primed’ state with TFs FLI, SCL and CEBPβ already bound and with RNA Polymerase II (Pol II) already present at many promoters^13^. It is widely accepted that TFs binding to enhancers direct the onset of transcription by recruiting co-activators, together with the mediator complex, which interact with the basal transcription machinery containing the positive transcription elongation factor b (pTEFb) complex. Activation of pTEFb by interaction with TFs, with the bromodomain protein BRD4 or the super elongation complex (SEC) causes phosphorylation of the Pol II CTD by the kinase component of this complex, CDK9, resulting in release of the polymerase from a paused state^14,15^. Mediated by bridging molecules such as LDB1, promoter and enhancer elements physically and differentially interact in nuclear space providing an additional layer of gene regulation^16,17^. We showed that RUNX1 directly orchestrates both processes since its induction rapidly activated the transcription of haematopoietic genes and re-shaped the epigenetic landscape by causing increased histone acetylation and global redistribution of TF complexes^13^. However, the precise nature and order of factors recruited by RUNX1 are still unclear.

In this study, we used the iRUNX1 system to dissect, at the global level, how RUNX1 orchestrates the formation of transcription factor complexes and Pol II recruitment to drive the transcriptional processes that underlie the EHT. We show that after induction, genome-wide redistribution of TF complexes by RUNX1 is coupled with increased enrichment of CDK9 at distal sites, the onset of transcription of genes essential for haematopoietic development and increased deposition of the H3K79me2 mark on histones as a marker for transcriptional elongation. In parallel, the bridging factor LDB1 moves with the other factors bound at distal sites towards RUNX1 bound sequences and leads to recruitment of the Mediator complex. Factor movement is blocked by treatment with the bromodomain inhibitor JQ1, leading to a block in the up-regulation of haematopoietic genes and the EHT. Our data show a direct requirement for RUNX1 to recruit factors associated with transcriptional elongation and to orchestrate the reorganisation of interacting transcription factor complexes, thus facilitating a permissive environment for the rapid activation of genes essential for the EHT.

## Results

### Induction of RUNX1 leads to increased histone acetylation and BRD4 recruitment at distal RUNX1 binding sites

**Figure 1:**
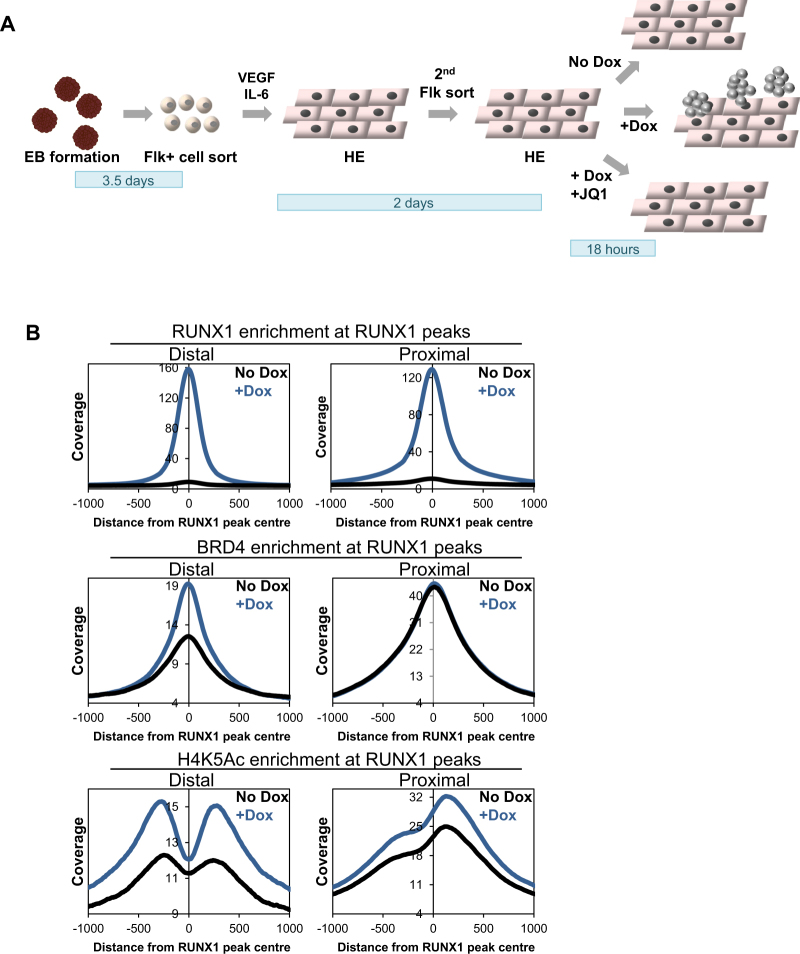
Induction of RUNX1 leads to increased BRD4 and histone acetylation at distal RUNX1 binding sites. (**A**) Schematic diagram depicting the *in vitro* differentiation system used in this study. FLK1+ haemangioblast cells were isolated from EBs and grown in blast culture media containing VEGF and IL-6 for 2 days. FLK1 expressing HE cells were then purified and cells were grown in HE media and treated with the indicated conditions for 18 hrs. (**B**) Average profiles of RUNX1 (Top panel), BRD4 (Middle panel) and H4K5Ac (Bottom panel) ChIP-seq peak enrichment centred on RUNX1 peaks (+/−1000 bp from peak centre). Enrichment at distal (left panels) and proximal (right panels) RUNX1 binding sites are shown.

The generation of blood cell progenitors from the haemangioblast via a haemogenic endothelium intermediate stage has been well characterised and surface marker expression identifying the different cell populations has been described^10^. Early haemogenic endothelium (HE1) expresses c-Kit and the endothelial marker Tie2 but is negative for CD41, a marker of haematopoietic commitment (HE1: c-Kit+, Tie2+, CD41−). A later stage of HE - HE2 - acquires CD41 expression as cells progress through differentiation (HE2: c-Kit+, Tie2+, CD41+). As cells progress further towards haematopoietic commitment they lose the Tie2 endothelial marker (Haematopoietic Progenitors (HP): c-Kit+, Tie2−, CD41+). In the absence of RUNX1 the doxycycline (Dox) inducible iRUNX1 ES cell line is unable to progress past the haemogenic endothelium (HE) stage of development during *in vitro* haematopoietic development (Fig. 1A)^10,13^.

To avoid inducing excessive amounts of RUNX1 we tested the minimum amount of Dox required to allow the EHT to progress and for CD41+ haematopoietic progenitors to form from Flk1 expressing HE cells. The numbers of cells expressing c-Kit, CD41 and Tie2 were measured by flow cytometry (Supplementary Fig. S1A). A graph representing the proportions of the cell populations as defined by these markers is shown in Supplementary Fig. S1B and together with Fig. S1A demonstrate a dose dependent increase in committed progenitors in response to RUNX1 induction. A small number of cells express Tie2 and CD41 in the absence of RUNX1 and these represent a population of primitive erythroid cells which are formed independent of RUNX1^10^. The proportional changes in surface marker expression of the cell populations were coupled with changes in gene expression as shown in Supplementary Fig. S1C, with increased expression of haematopoietic genes *Gfi1* and *Spi1* and decreased expression of HE-expressed *Sox17* in the Dox treated samples. The optimal requirement for changes in gene expression was 0.1 μg/ml Dox. The level of RUNX1 protein induced is shown in Supplementary Fig. S1D.

To explore the molecular mechanism underpinning the rapid genomic and cellular response to RUNX1 induction, we investigated the basal and tissue-specific transcriptional regulators co-operating with RUNX1 driving the EHT in ES cell derived haemogenic endothelium cells. RUNX1 is known to interact with the transcriptional co-activators p300 and CBP leading to increased histone acetylation and acetylation of RUNX1 itself^18,19^. The bromodomain protein BRD4 binds acetylated histones, including the H4K5Ac histone mark, as well as acetylated TFs^20,21^ and promotes transcriptional elongation by the recruitment of the Mediator complex and pTEFb^21^. In order to examine whether these factors respond to RUNX1 induction at its targets, we first examined their binding by chromatin immunoprecipitation followed by next generation sequencing (ChIP-seq) of RUNX1, BRD4 and the histone modification H4K5Ac in HE cells which were either untreated (No Dox) or Dox treated (+Dox). RUNX1 bound at 18,430, mostly distal, peaks and correlated with the presence of RUNX motifs (Supplementary Fig. S1E). Average profiles of RUNX1, BRD4 and H4K5Ac at RUNX1 binding sites demonstrated that although BRD4 was already present in the absence of RUNX1, BRD4 binding was specifically increased at distal but not at the proximal RUNX1 binding sites following RUNX1 induction, whereas H4K5Ac was increased at both (Fig. 1B), indicating that (i) promoters and distal RUNX1 target sites were already primed and that (ii) RUNX1 induction increased recruitment of BRD4 to distal but not proximal sites.

### Inhibition of BRD4 blocks the endothelial-haematopoietic transition

**Figure 2:**
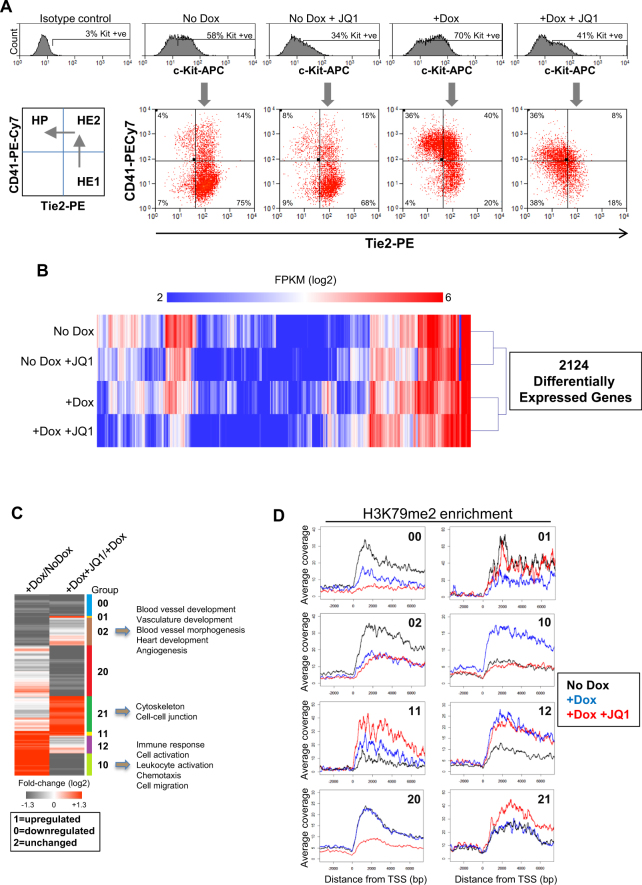
Perturbation of the EHT by the bromodomain inhibitor JQ1. (**A**) Representative flow cytometry analysis from four independent experiments showing the effect of treatment with JQ1 on the differentiating cell populations. Adherent cells from each of the treatment conditions were stained with c-Kit-APC, CD41-PECy7 and Tie2-PE. Populations were gated on c-Kit positive cells (upper histogram) and cells within this gate expressing CD41 and Tie2 are shown in the quadrant plot below. The bottom left panel indicates the cell populations represented in each of the quadrants of cells stained with c-Kit-APC, CD41-PECy7 and Tie2-PE and also indicates the direction of differentiation. (**B**) Heatmap showing hierarchical clustering of log2 FPKM for the 2124 genes that change expression at least 2 fold between treatment conditions as defined in Supplementary Fig. 2B. (**C**) Changes in gene expression were grouped according to whether genes were up-regulated (1), down-regulated (0) or unchanged (2) between the indicated treatment conditions. The number of the group relates to changes between the +Dox compared to the No Dox sample (1^st^ column) and changes between the +Dox +JQ1 compared to the +Dox sample (2^nd^ column). GO terms for selected groups are indicated on the right hand side of the figure. See related Figure S2D for numbers of genes within each group. (**D**) Average profile of H3K79me2 enrichment for each of the 8 gene expression groups shown in Fig. 2C.

BRD4 has been reported to contribute to recruitment of the pTEFb complex^22^. To investigate the role of BRD4 in RUNX1-mediated changes in gene expression and factor recruitment, we modulated its activity using the bromodomain inhibitor JQ1^23^. A number of studies indicated that the effect of JQ1 inhibition is context dependent suggesting that BRD4 plays multiple and highly diverse roles in transcriptional regulation^24–26^. To examine the role of BRD4 in RUNX1-mediated transcription, we studied the effect of JQ1 treatment on the formation of haematopoietic progenitors in the presence and absence of RUNX1 induction using flow cytometry analysis after 18 hour treatment with Dox and JQ1 (Fig. 2A). While JQ1 alone reduced the number of cells expressing c-Kit, CD41 and Tie2 expression were relatively unaffected within the c-Kit positive population. In Dox-induced cells, the number of cells co-expressing CD41 and c-Kit but not Tie2 increased as expected indicating differentiation to haematopoietic progenitors. Cells treated with both Dox and JQ1 at the same time showed a reduction in c-Kit expressing cells and reduced staining intensity of CD41 and Tie2. The expression of genes normally up-regulated by RUNX1, such as *Gfi1* and *Spi1*, was reduced in the presence of JQ1 reflecting the inhibition of the EHT and the reduced formation of haematopoietic progenitors (Fig. S2A).

Global analysis of gene expression by RNA-Seq showed that JQ1 had a dramatic influence on RUNX1 induced transcription. More than 2,000 genes were deregulated across the treatment conditions, with the highest number of down-regulated genes observed between the +Dox and +Dox +JQ1 samples (Supplementary Fig. S2B and Supplementary Table S1) indicating that JQ1 strongly interfered with RUNX1-mediated gene expression changes. Analysis of the 4 different treatment conditions showed that each treatment led to widespread changes in gene expression as shown by hierarchical clustering of the log2 FPKM values of the differentially expressed genes (Fig. 2B). Clustering analysis of the Pearson correlation coefficient showed that each of the conditions clustered as distinct groups and replicates clustered together (Supplementary Fig. S2C). We next focused on the gene expression changes between Dox-induced cells with and without JQ1 and the No Dox control cells. Gene expression patterns could be separated into 8 groups based on whether genes were upregulated (1), downregulated (0) or unchanged (2) in the +Dox sample compared to the No Dox sample and then upregulated, downregulated or unchanged in the +Dox +JQ1 sample compared to the +Dox sample (Fig. 2C and Supplementary Fig. S2D). For example, Group 10 relates to genes normally up-regulated by RUNX1 but which are inhibited by JQ1 treatment. This group includes genes such as *Myb*, *Spi1*, *Gfi1* and *Vav1* and includes gene ontology (GO) terms related to haematopoiesis, cell migration and adhesion (Fig. 2C, Supplementary Table S2). *Myb* is essential for definitive haematopoiesis^27^ and *Vav1* is a GDP/GTP nucleotide exchange factor (GEF) which is expressed at early stages of the haematopoietic system and is involved in reorganisation of the cytoskeleton amongst other functions^28^. Genes normally strongly down-regulated by RUNX1 exhibit a varied response to JQ1 (Fig. 2C, Groups 00, 01 and 02). However genes remaining repressed following JQ1 treatment (Group 02) score highly for gene ontology terms relating to blood vessel and cardio-vasculature development and include *Notch 1* and several Rho guanine nucleotide exchange factors (RhoGEFs), indicating that cells adopt a fate that is related to that of endothelial cells, again confirming that the EHT did not take place.

The histone modification H3K79me2 is deposited along the gene body during transcription and is recognised as a mark of transcriptional elongation^29,30^. We analysed the association between the 8 different gene expression clusters and the H3K79me2 mark and found that the change in H3K79me2 enrichment across the gene body reflected the changes in gene expression, indicating that the response to RUNX1 induction is indeed transcriptional (Fig. 2D). Using Group 10 as an example, we find that the average profile of H3K79me2 enrichment showed increased levels of this histone mark across the gene body in the +Dox sample compared to the No Dox and +Dox +JQ1 sample (Fig. 2C and Fig. 2D), indicating that transcription of these genes is sensitive to JQ1 treatment. In contrast, Group 12 genes also show increased expression and H3K79me2 enrichment but their expression is unchanged in response to JQ1 treatment and H3K79me2 enrichment persists at the gene body.

Our results indicate that induction of RUNX1 is associated with increased transcriptional elongation at a subset of genes and that JQ1 treatment blocks transcription of a proportion of these genes resulting in a failure of the EHT.

### BRD4 inhibition disrupts RUNX1 binding and CDK9 recruitment at a subset of genes

**Figure 3:**
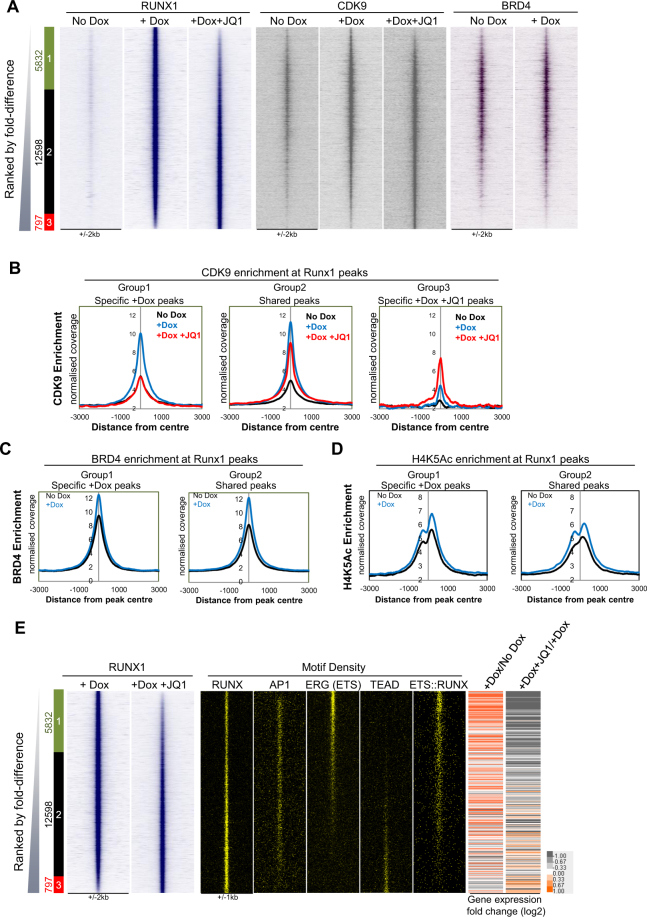
Treatment with JQ1 inhibits RUNX1 binding and CDK9 recruitment to specific sites. (**A**) Heat maps showing ChIP-seq enrichment of RUNX1 binding for the No Dox, +Dox and +Dox +JQ1 samples, ranked according to fold difference between +Dox and +Dox +JQ1 samples. Peaks were considered to be specific if they showed greater than 2 fold enrichment in one sample compared to the other. The specific/shared groups and the numbers of peaks within these groups are shown alongside. Group 1 (green box) are +Dox-specific RUNX1 bound peaks; Group 2 (black box) are occupied by RUNX1 in both samples and Group 3 (red box) are +Dox +JQ1-specific RUNX1 bound peaks. Ranked along the same coordinates are heatmaps showing CDK9 enrichment for the No Dox and +Dox samples with and without JQ1 and BRD4 enrichment for the No Dox and +Dox samples. (**B**) Average profiles of CDK9 enrichment at the specific and shared RUNX1 bound sites for each of the different treatment conditions. The profiles were centred on RUNX1 peaks (+/−3000 bp from peak centre) and are shown for the specific and shared groups assigned in Fig. 3A. (**C**) Average profiles of BRD4 enrichment centred on RUNX1 peaks (+/−3000 bp from peak centre) in the shared and specific RUNX1 peaks from Fig. 3A. (**D**) Average profiles of H4K5Ac enrichment centred on RUNX1 peaks (+/−3000 bp from peak centre) in the shared and specific RUNX1 peaks from Fig. 3A. (**E**) Heat maps showing RUNX1 enrichment for the +Dox and +Dox +JQ1 samples (as shown in Fig. 3A). Shown alongside are motif density plots for the RUNX, AP1, ERG (ETS), TEAD and ETS::RUNX composite motifs. Also shown are heatmaps showing gene expression fold changes between +Dox compared to No Dox and +Dox +JQ1 compared to +Dox samples for the genes attributed to the corresponding RUNX1 ChIP-seq peaks.

To examine the molecular mechanism of the block in the transcription of genes essential for the EHT after JQ1 treatment, we studied factor recruitment events at direct RUNX1 target sequences by performing ChIP-seq for RUNX1 in un-induced and induced cells in the presence and absence of JQ1 (Fig. 3A). We then ranked the peaks according to the fold difference between RUNX1 binding with and without JQ1 and peaks were considered to be specific if they showed more than two fold enrichment in one condition compared to the other. As expected there were no significant peaks identified in un-induced cells. In the Dox-induced cells RUNX1 bound 18,430 sites which was reduced to 13,395 peaks in the +Dox +JQ1 cells (Supplementary Fig. S3A). The distribution of RUNX1 binding sites between promoter, intragenic and intergenic regions was similar (Supplementary Fig. S3A) and the overlap of the peaks from the +Dox and +Dox +JQ1 cells revealed a large number of shared peaks (Supplementary Fig. S3B). In the presence of JQ1, RUNX1 binding was reduced greater than 2-fold or absent at 5832 peaks (Group 1, Fig. 3A). This reduction was not a global response since two thirds of RUNX1 binding sites were unaffected and *de novo* RUNX1 binding was acquired at 797 sites.

We next plotted heatmaps of CDK9 and BRD4 ChIP-seq peaks for the same RUNX1 peaks to give a RUNX1-centric view of CDK9 and BRD4 enrichment at these sites (Fig. 3A). The corresponding CDK9 enrichment at RUNX1 target sites increased at both shared and specific RUNX1 bound sites in the +Dox sample (Fig. 3A,B). The sites unable to bind RUNX1 after JQ1 treatment also lost CDK9 binding (Fig. 3A,B, Group 1). Conversely, the newly acquired RUNX1 binding sites in the +Dox +JQ1 treated cells gained CDK9 enrichment (Fig. 3A,B, Group 3), indicating a close association between RUNX1 and CDK9 but also highlighting that this interaction was context specific. BRD4 was already present in un-induced cells at many of the specific and shared sites where RUNX1 would subsequently bind (Fig. 3A,C). There was a modest increase in both BRD4 and H4K5Ac at both shared and specific RUNX1 binding sites (Fig. 3C,D), however, co-immunoprecipitation of RUNX1 and BRD4 failed to reveal a direct interaction between these proteins (Supplementary Fig. S3C), indicating that BRD4 is recruited by a different factor.

We analysed the RUNX1 binding sites in more detail by performing TF motif analysis and integrating the gene expression changes of RUNX1 target genes (Fig. 3E, Supplementary Fig. S3D and Supplementary Table S3). We found that the most JQ1 sensitive RUNX1 binding sites were highly enriched for ETS or ETS::RUNX composite motifs and that these sites corresponded to genes that were up-regulated by RUNX1 induction and down-regulated in response to JQ1 (Fig. 3E and Supplementary Fig. S3E). Conversely, the newly acquired RUNX1 binding sites in the +Dox +JQ1 sample that were associated with genes up-regulated following JQ1 treatment were enriched for the TEAD motif which is a hallmark of vascular cells^31^. These results again show that JQ1 treated cells adopt a different identity, suggesting that differential sensitivity of RUNX1 binding to JQ1 inhibition is context dependent.

### BRD4 inhibition interferes with the relocation of FLI1 towards RUNX1 binding sites

**Figure 4:**
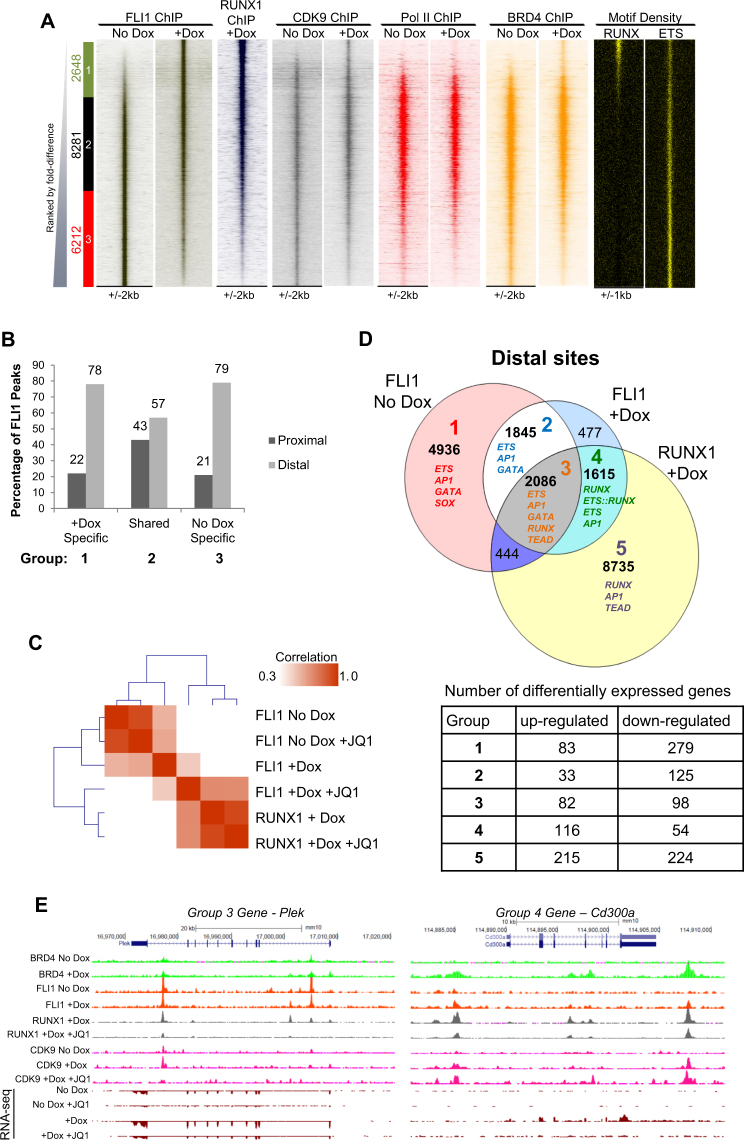
Recruitment of CDK9 to distal sites by RUNX1 and FLI1. (**A**) Comparison of FLI1 binding in ChIP-seq from No Dox and +Dox treated samples ranked according to fold difference. Peaks were considered to be specific if they showed greater than 2 fold enrichment in one sample compared to the other and are designated as Group1, 2 or 3. The specific/shared groups and the numbers of peaks within these groups are shown alongside. Group 1 (green box) are +Dox-specific FLI1 bound peaks; Group 2 (black box) are occupied by FLI1 in both samples and Group 3 (red box) are No Dox-specific FLI1 bound peaks. RUNX1 binding in the +Dox treated samples, CDK9, BRD4 and Pol II ChIP-seq enrichment in the No Dox and +Dox samples and the RUNX and ETS motif density plots are ranked along the same coordinates. (**B**) Genomic distribution of FLI1 binding sites in the specific and shared sites for Fig. 4A. Percentages of proximal and distal sites are shown above the bars. (**C**) Clustering analysis based on the Pearson correlation for the RUNX1 and FLI1 ChIP-seq samples. (**D**) Venn diagram showing the peak overlap between FLI1 binding in the No Dox and +Dox samples and RUNX1 binding in the +Dox sample. Five groups are highlighted based on these overlaps and the diagram is annotated with the most highly represented TF motifs for each of the groups 1–5 and the number of peaks within each group. The table below shows the number of differentially expressed genes relating to each group. Differentially expressed genes are those which change expression more than 2 fold between the +Dox compared to the No Dox sample as shown in Supplementary Fig. S2B. (**E**) Genome browser screenshots depicting the indicated ChIP-seq tracks for *Plek* and *cd300a* as representative genes from Groups 3 and 4 respectively, from Fig. 4D.

The strongest inhibition of RUNX1 binding by JQ1 correlated with down-regulation of gene expression of the nearest genes to the RUNX1 peak and a strong enrichment of ETS motifs at the regions normally bound by RUNX1 (Fig. 3E). Expression of the ETS family transcription factor FLI1 precedes that of RUNX1 and is essential for the normal development of blood vessels and haematopoiesis^32^. Our previous work showed that RUNX1 induction in these cells results in the redistribution of TF complexes containing FLI1^13^. We therefore sought to determine whether JQ1 blocked the EHT by preventing this redistribution and the formation of new TF complexes. We first performed a pairwise comparison of FLI1 binding (FLI1-centric view) with and without RUNX1 induction and found that FLI1 binding increased more than two fold at 2648 sites (Group 1, Fig. 4A). The comparison with RUNX1, CDK9 and Pol II ChIP enrichment plotted alongside showed that these peaks overlapped with RUNX1 sites that acquired CDK9 as well as BRD4 following RUNX1 induction. FLI1 binding was reduced or lost at 6212 sites. These sites showed low or no RUNX1 binding and lost CDK9 and BRD4 enrichment (Group 3, Fig. 4A). Interestingly, the peaks within Groups 1 and 3 contained low levels of Pol II and subsequent analysis of their genomic location found that both sets of unique peaks comprised almost 80% distal sites compared to the shared sites which contained 43% proximal and 57% distal sites (Fig. 4B). This result is supportive of the concept of distal enhancers, rather than gene promoters, being associated with changes in occupancy during differentiation and lineage specification. In this case, this includes the binding of the basal transcription machinery in the form of CDK9 which follows FLI1 and RUNX1.

To examine the effect of JQ1 treatment on FLI1 binding we performed ChIP-seq for FLI1 in RUNX1 induced and un-induced cells in the presence and absence of JQ1 and ranked the tag counts alongside those of the RUNX1 ChIP data (RUNX1-centric view). Similar numbers and distribution of peaks were observed in each of the samples (Supplementary Fig. S4A,B) indicating that FLI1 binding was not generally inhibited. We found that the FLI1 binding patterns were similar in the absence of RUNX1 even with JQ1 treatment (Supplementary Fig. S4A), whereas in +Dox treated cells FLI1 binding was altered and followed a similar pattern to RUNX1 binding (Supplementary Fig. S4A). Clustering analysis of the FLI1 bound sequences under the 4 conditions together with the RUNX1 peaks confirmed that in the absence of Dox, FLI1 peaks cluster closely together, however following Dox induction, the correlation is weaker and the percentage of FLI1 peaks associated with RUNX1 peaks increases (Fig. 4C and Supplementary Fig. S4C). Interestingly, the +Dox +JQ1 sample clusters even more closely suggesting that FLI1 co-occupies even more RUNX1 sites in these aberrant cells.

In order to explore the underlying TF binding motif landscape mediating the binding of RUNX1 and FLI1 we overlapped FLI1 distal peaks in induced and un-induced cells and RUNX1 distal peaks. We then identified the most enriched TF motifs for each of the indicated Groups 1–5 shown on the Venn diagram in Fig. 4D, together with the number and behaviour of associated genes (lower panel). The full motif table for Groups 1–5 is shown in Supplementary Fig. S4D and genes for these groups are shown in Supplementary Table S4. This analysis demonstrates that in the absence of RUNX1, FLI1 mainly binds at ETS sites that are associated with GATA and AP1 motifs (Groups 1 and 2, Fig. 4D and Supplementary Fig. S4D). After RUNX1 induction and binding, a proportion of the FLI1 sites remain bound at a subset of sites that contain ETS, AP1, RUNX, TEAD and a lower proportion of GATA motifs (2086 genes, Group 3, Fig. 4D and Supplementary Fig. S4D).

A further 1615 *de novo* peaks appear at sites which harbour RUNX, ETS or ETS::RUNX composite motifs and to a lesser extent AP1 motifs (Group 4, Fig. 4D and Supplementary Fig. S4D). Group 3 includes peaks related to genes associated with haematopoiesis such as *Gfi1* and genes related to actin reorganisation and integrin signalling such as *Plek* and *Vav1*. Group 4 also includes genes related to cytoskeletal organisation such as *Ccl3* and *Diaph1* and immune system genes such as *Cd300a*. Group 5 contains mainly RUNX1 specific bound peaks and this is reflected in the lack of ETS motif in this group (Group 5, Fig. 4D and Supplementary Fig. S4D). Screenshots of example genes from Groups 3 and 4 are shown in Fig. 4E, illustrating the changes in FLI1, RUNX1 and CDK9 occupancy at distal sites in the different conditions. The plots of the average profiles of CDK9 enrichment for Groups 1–5 demonstrate an increased recruitment of CDK9 in Groups 3–5 following RUNX1 induction and binding (Supplementary Fig. S4E). Peaks at many genes including *Vav1* and *Cd300a* are represented in more than 1 group, indicating the complex regulatory mechanisms that exist for the control of lineage decision making processes. Our ChIP-seq data therefore demonstrate that in addition to binding to a large number of sites alone, RUNX1 also forms complexes containing FLI1, together with CDK9. Whether FLI1 changes its binding location appears to depend on the underlying motif of the region it binds. Overall, RUNX1/FLI1 complexes move away from ETS/GATA motifs and towards RUNX/ETS motifs as cells differentiate from early haemogenic endothelium towards haematopoietic progenitors.

### RUNX1 recruits LDB1 to distal sites

**Figure 5:**
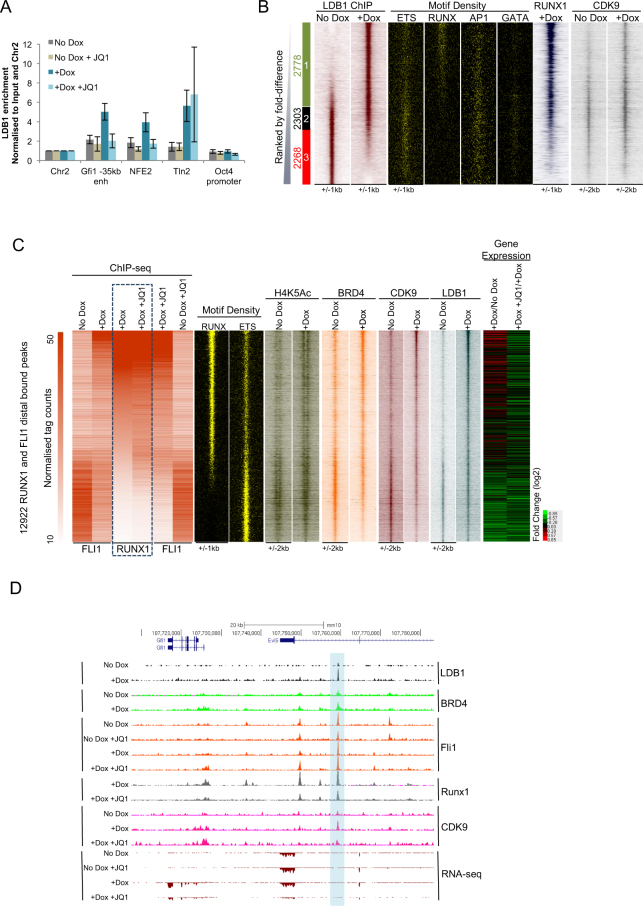
RUNX1 recruits LDB1 and co-ordinates TF assembly at genes essential for the EHT and haematopoiesis. (**A**) Manual LDB1 ChIP showing recruitment of LDB1 to regulatory elements known to be bound by RUNX1. Error bars represent standard deviation, n = 3. (**B**) Heat maps showing enrichment of LDB1 ranked by fold difference between the +Dox and No Dox samples. Peaks were considered to be specific if they showed greater than 2 fold enrichment in one sample compared to the other and are designated as Group1, 2 or 3. The specific/shared groups and the numbers of peaks within these groups are shown alongside. Group 1 (green box) are +Dox-specific LDB1 bound peaks; Group 2 (black box) are occupied by LDB1 in both samples and Group 3 (red box) are No Dox-specific LDB1 bound peaks. Motif density plots for RUNX, ETS, AP1 and GATA motifs and ChIP-seq enrichment for RUNX1 and CDK9 at these sites are also shown along the same coordinates. (**C**) Heat maps showing RUNX1 (highlighted with dashed line) and FLI1 ChIP-seq normalised tag counts for distal sites bound by RUNX1 and/or FLI1 ranked according to the strength of RUNX1 binding. Also shown for the same regions are: RUNX and ETS motif density plots; H4K5Ac, BRD4, CDK9 and LDB1 ChIP-seq enrichment for these distal sites and gene expression for genes attributed to these sites. (**D**) Genome browser tracks showing the LDB1, BRD4, FLI1, RUNX1 and CDK9 ChIP-seq enrichment and gene expression at the *Gfi1* locus. The shaded region highlights the Gfi1 −35 kb enhancer used for validation in Fig. 5A.

The LDB1 protein has been identified as essential for the differentiation of the erythroid lineage and forms a bridging complex with GATA, TAL1 and KLF factors^17,33^. It is sufficient to coordinate the interaction of distal elements with their promoters^34^. Moreover, a crucial role for LDB1 has previously been shown in ES cell differentiation into blood^35^. We therefore used manual ChIP to test whether LDB1 was present at enhancer regions in our differentiation system (Fig. 5A). The *Gfi1* -35kb enhancer and the *NFE2* −3 kb regulatory element both showed specific enrichment of LDB1 which was absent in un-induced cells and in the presence of JQ1. However, the response of LDB1 to JQ1 treatment was again context dependent, as it was still bound at a distal regulatory element within the *Tln2* gene following JQ1 treatment, corresponding to increased expression of *Tln2* in the +Dox +JQ1 sample. We found a similar complex pattern of ChIP enrichment for the Mediator complex subunit Med12 (Supplementary Fig. S5A). To examine how LDB1 binding related to global RUNX1 binding, we performed ChIP-seq of LDB1 before and after RUNX1 induction and plotted the binding sites alongside RUNX1, CDK9 and relevant binding motifs (Fig. 5B). While we found LDB1 binding at ETS sites prior to RUNX1 induction, it became strongly associated with RUNX1 sites and increased CDK9 enrichment after induction. This was confirmed by motif analysis of the LDB1 ChIP-seq peaks. RUNX motifs were absent prior to Dox induction in the un-induced sample, however RUNX motifs were present in 30% of sites after induction (Supplementary Fig. S5B).

To display a comprehensive picture of how complexes form at distal sites in response to RUNX1 induction we integrated all of our ChIP-seq data with motif density plots and gene expression data. In Fig. 5C we ranked the normalized tag counts for each ChIP experiment alongside the induced RUNX1 peaks in the presence and absence of JQ1. FLI1, H4K5Ac, BRD4, CDK9 and LDB1 all move towards the RUNX1 bound regions following Dox treatment. Treatment with JQ1 in the presence of RUNX1 (+Dox +JQ1) causes a further redistribution of FLI1 binding that does not occur in the absence of Dox, suggesting that RUNX1 informs the FLI1 binding pattern even under different stimuli. An example for the dynamics of TF recruitment after RUNX1 induction (the *Gfi1* locus) is shown in Fig. 5D. In this case, RUNX1 binds to the −35 kb distal enhancer (shaded region, Fig. 5D) and despite FLI1 already being bound at this location, it is only upon RUNX1 induction that LDB1 is enriched at this site. RUNX1 binding also correlates with increased CDK9 enrichment at this distal site and a corresponding increase in *Gfi1* expression as detected by RNA-seq. The gene ontology terms associated with the RUNX1 bound up-regulated genes – within the top half of the heatmap in Fig. 5C - relate to haematopoiesis and blood cell activation and migration (Supplementary Fig. S5C and Supplementary Table S5 for GO terms) while genes in the bottom half of the heatmap in Fig. 5C represent down-regulated FLI1 targets and relate to angiogenesis and vasculature development (Supplementary Fig. S5D and Supplementary Table S5 for GO terms) again highlighting the switch in cell fate initiated by RUNX1. In summary, our data show a direct requirement for RUNX1 to recruit factors associated with transcriptional elongation and to reorganize interacting transcription factor complexes, thus facilitating a permissive environment for activation of genes essential for the EHT. Our data also highlight the breathtaking complexity and dynamics of how transcription factors and co-factors are capable of rapid assembly and de-assembly during the replication-independent transition of one cell fate to another.

## Discussion

**Figure 6:**
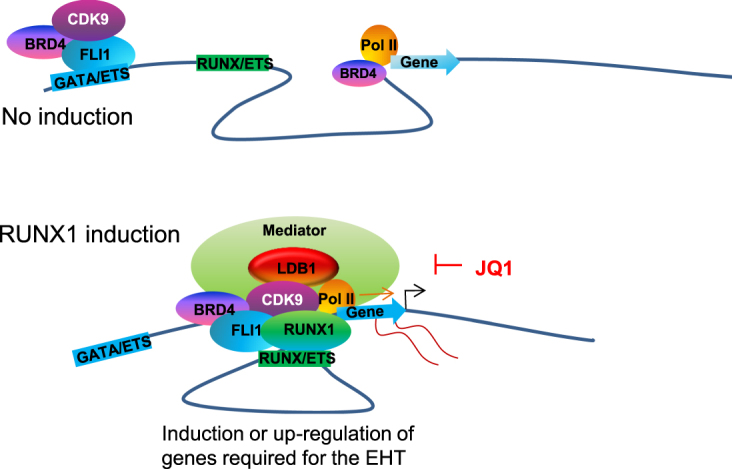
Model depicting RUNX1 mediated recruitment of the pTEFb complex and LDB1 resulting in the onset of gene expression at RUNX1 target genes. Following induction of RUNX1 expression by Dox treatment, FLI1 and BRD4 move closer to RUNX1 sites which is associated with increased histone acetylation, CDK9 and LDB1 binding and activation of expression of haematopoietic genes.

Using an inducible RUNX1 ES cell line we previously found that RUNX1 orchestrates the relocation of the transcription factors FLI1 and SCL/TAL1 during the EHT^13^. In our current study, we have used the same system to identify the molecular details of how the reshaping of the transcription factor binding landscape links to the rapid initiation of active transcription at lineage specific genes. From this data we can build a model of how RUNX1 orchestrates the formation of transcription factor complexes at distal sites of genes up-regulated during the EHT (Fig. 6). In the absence of RUNX1, FLI1 binds at ETS sites associated with nearby GATA and AP1 motifs that are characterized by low levels of BRD4 binding. Such primed binding sites are associated with genes regulating haematopoiesis and cell shape changes.

Following induction of RUNX1, and in parallel with changing cell shape, factors rapidly re-locate within the genome. Our data show that either FLI1 moves to ETS sites close to RUNX1 enriched in composite ETS/RUNX motifs, or RUNX1 binds close to sites where FLI1 is already bound. These movements in transcription factor binding are associated with recruitment of LDB1, BRD4 and CDK9 to distal sites, increased histone acetylation and corresponding changes in gene expression. The correlation between TF movements and LDB1/mediator recruitment to sites binding RUNX1 and FLI1 suggests that promoter and distal enhancer regions are brought together to facilitate transcription.

Prior to RUNX1 induction CDK9 binds to distal sites of genes essential for the haematopoietic lineage such as *Spi1*, *Gfi1* and *Myb*. Treatment with the bromodomain inhibitor JQ1 blocks the RUNX1 induced movement of FLI1/CDK9 towards sites associated with genes driving the EHT and RUNX1/LDB1/Mediator binding. JQ1 treatment leads to the movement of RUNX1 and FLI1 to alternative sites and the formation of an aberrant cell type unable to conduct the EHT, indicating that factor relocation, complex assembly and cell differentiation are intricately coupled. However, the inhibition of the redistribution of FLI1 (and most likely also other factors) towards RUNX1 does not involve binding of BRD4 to RUNX1, as we were unable to see an interaction between RUNX1 and BRD4. JQ1 affected CDK9 recruitment but only at sites where RUNX1 recruitment was lost as well. Roe *et al*. found that in addition to acetylated histones, BRD4 could interact with acetylated TFs including FLI1 (RUNX1 was not studied)^21^. Our ChIP-seq data support this mechanism (Fig. 5C), with BRD4 enrichment coinciding with, and following, FLI1 binding and with H4K5Ac enrichment predominantly flanking the TF binding sites (Fig. 5C). RUNX1 and FLI1 directly interact^13^, our data are therefore consistent with the idea that RUNX1 pulls in a FLI1/BRD4 complex. In addition, RUNX1 is known to interact with the transcriptional co-activators p300 and CBP leading to increased histone acetylation and acetylation of RUNX1 itself^18,19^. We have shown that induction of RUNX1 leads to a widespread increase in H3K9Ac at target gene loci^13^, as well as H4K5Ac (this study) which could further stabilize BRD4 binding to RUNX1 binding sites.

Transcription factor binding at enhancers or promoters is determined by the underlying DNA sequence. We found that the movement of TF complexes mediated by RUNX1 follows a specific binding motif code. In the absence of RUNX1, FLI1 binds to sites with ETS motifs together with AP1 and GATA motifs. Following RUNX1 induction around 2000 of these sites are retained, however, a substantial proportion of *de novo* FLI1 binding sites are observed (1615) and these contained RUNX or RUNX::ETS composite motifs. This result would suggest that co-operative binding of RUNX1 and FLI1 is essential for progression of the EHT and that changes in the composition of TF complexes, e.g. loss of GATA motif binding factors and gain of RUNX1, are required to drive such cell shape switches. The binding of different combinations of factors to different cis-regulatory elements is associated with cell lineage specification^1,2^. Our study provides a direct mechanism for how the presence or absence of one factor differentially drives such changes.

Mylona *et al*. demonstrated a function for LDB1 early in blood cell development where they showed defective blast-colony formation from Flk1+ haemangioblast cells from LDB1−/− ES cells. Many of the pathways deregulated in the absence of LDB1 were also deregulated by blocking the EHT with JQ1 treatment, including genes involved in integrin signalling, focal adhesion and the Wnt pathway^35^. LDB1 has been shown to function by mediating long-range promoter-enhancer interactions^17^. Based on their observations in the Myb locus^36^ where multiple enhancers control tissue specific expression of the *myb* gene, Stadhouders *et al*. proposed a model for gene activation involving the looping of distal element-bound CDK9 to genic regions^16^. RUNX1 has been shown to mediate cis-regulatory element interactions at the human CD34 locus^37^. Together with the strong global association between RUNX1 binding, FLI1/BRD4 relocation, CDK9 and LDB1 enrichment at RUNX1 target genes this suggests that RUNX1 associated transcription factor assemblies mediate the initiation of transcriptional elongation through looping.

We suggest that such specific and rapid factor relocation processes mediated by protein-protein interactions, as described here, drive all replication-independent transitions in cellular shape and function that are widely observed in development. It is important to note that our findings are also relevant in a disease context. A number of haematopoietic malignancies are caused by mutations in *RUNX1*, in particular in acute myeloid leukemia (AML). For example, in t(8; 21) AML the DNA binding domain of RUNX1 is fused to the repressor ETO (MTG8) turning this protein into a constitutive repressor, causing a block in differentiation at an early myeloid progenitor stage^38^. Structural and functional studies demonstrated that the fusion protein exists in a complex containing FLI1 and LDB1^39–41^ and knock-down of the fusion protein leads to cell differentiation and changes in the binding patterns of myeloid transcription factors^42^. Conversely, induction of the fusion protein in the haemogenic endothelium blocks the EHT and leads to a block in differentiation when induced in progenitors^43^. It is therefore likely that the fusion protein is unable to orchestrate the regulated movement of transcription factor complexes described here.

In summary, the replication independent rapid change in cell shape during the endothelial-haematopoietic transition (EHT) starts out from a primed state and involves the RUNX1-dependent recruitment of BRD4, CDK9, LDB1 and Mediator to RUNX1 binding sites at genes required for haematopoietic differentiation. The formation of transcriptionally competent TF complexes at RUNX1 target genes requires the relocation of BRD4-FLI1 complexes from GATA/ETS to RUNX1/ETS sites. Inhibition by the BRD4 inhibitor JQ1 blocks formation of these complexes, thus preventing the EHT and haematopoietic development.

## Methods

### ES cell culture

The iRUNX1 ES cell line was maintained as described previously^13,43^. This cell line carries an HA-tagged RUNX1 under the control of a doxycycline inducible promoter in a RUNX1 null background^10^. Briefly, cells were grown on mouse embryonic feeder cells (MEFs) inactivated with mitomycin C (Sigma), in DMEM supplemented with 15% FCS (Stem CellTechnologies), 1 mM sodium pyruvate, 1 mM glutamine, 100 units per ml penicillin and 100 μg per ml streptomycin, 25 mM HEPES buffer, 1 × non-essential amino acids, 0.15 mM MTG and 10^3^ units per ml LIF (ESGRO mLIF, Millipore ESG1107).

### ES cell differentiation and JQ1 treatment

*In vitro* haematopoietic differentiation of the iRUNX1 ES cell line was performed essentially as described in Lichtinger *et al*.^13^. A more detailed description can be found in Supplementary Materials.

### Flow Cytometry

Haemogenic endothelium cells treated with and without Dox in the presence or absence of JQ1 were isolated for flow cytometry by trypsinising adherent cells, and washing with PBS, followed by MACS buffer. Cells were stained with antibodies against c-Kit (CD117-APC, BD Pharmingen 553356), CD41-PECy7 (eBioscience 25-0411), Tie2-PE (eBioscience 12-5987). Samples were run on a Cyan Flow Cytometer and analysed using Summit software (Beckman Coulter).

### Chromatin immunoprecipitation and library preparation

ChIP was performed as described previously^31^. ChIP-seq libraries were prepared using the Kapa Hyper Prep kit for Illumina platforms according to manufacturer’s instructions. More details including antibodies used and primer sequences for manual ChIP can be found in Supplementary Materials.

### RNA expression analysis and RNA-seq library preparation

RNA was extracted as described previously^44^, and more details including primer sequences for manual qRT-PCR can be found in Supplementary material. RNA-seq libraries were prepared from at least two biological replicates for each sample using the Tru-seq Stranded Total RNA kit (Illumina), according to manufacturer’s instructions. Libraries were sequenced in a pool of 12 indexed libraries using a NextSeq® 500/550 High Output Kit v2 (150 cycles) for paired end sequencing (Illumina, FC-404-2002) at the Genomics Birmingham sequencing facility.

### Co-immunoprecipitation assay and Western blot

Co-IPs were performed using the Pierce Crosslink Immunoprecipitation Kit (Thermo Fisher) according to manufacturer’s instructions. Samples were subsequently run on 4–20% gradient gels (Bio-Rad) and transferred to nitrocellulose membranes using a BioRad Trans-Blot Turbo transfer system. Membranes were blocked in 5% milk powder in 0.05% TBS-Tween and incubated with anti-HA (for RUNX1) (Sigma, H6908), anti-BRD4 (Bethyl Labs, A301-985A-100) and anti-CBFβ (Santa Cruz, sc20693) antibodies. Proteins were visualised using Pierce SuperSignal West Pico Chemiluminescent substrate (Thermo Scientific).

Western blots showing dose response of RUNX1 protein to Dox were performed as above then protein was detected using primary antibodies directed to AML1 (RUNX1) (Calbiochem, PC284) or GAPDH (Abcam, ab8245) followed by secondary antibodies IRDye® 800CW Donkey anti-Rabbit IgG (Li-Cor Biosciences) and IRDye® 800CW Donkey anti-Mouse IgG (Li-Cor Biosciences) respectively. Proteins were visualised on a Li-Cor Odyssey Imaging system.

### Data analysis

Methods used to analyse ChIP sequencing and RNA sequencing data are described in the Supplementary Methods.

### Availability of data

Data were deposited to NCBI under GSE104046.

## Electronic supplementary material

Supplemental Material

S1

S2

S3

S4

S5

Original blots
