# Supplementary material for: The Co-operation of RUNX1 with LDB1, CDK9 and BRD4 Drives Transcription Factor Complex Relocation During Haematopoietic Specification
Source: Sci Rep. 2018 Jul 10;8:10410. doi: 10.1038/s41598-018-28506-7 (PMC6039467; doi:10.1038/s41598-018-28506-7)
Supplement: Supplementary file 1 — Supplemental Material [file 41598_2018_28506_MOESM1_ESM.docx]

**The Co-operation of RUNX1 with LDB1, CDK9 and BRD4 Drives Transcription Factor Complex Relocation During Haematopoietic Specification.**

**Jane Gilmour^1^*, Salam A. Assi^1^, Laura Noailles^1^, Monika Lichtinger^1^, Nadine Obier^1,2^, and Constanze Bonifer^1^*.**

1 - Institute of Cancer and Genomic Sciences, College of Medical and Dental Sciences, University of Birmingham, Birmingham, B15 2TT, UK.

2 – Current address: Centre for Clinical Research, University of Freiburg Medical School, Freiburg, Germany.

*Corresponding Authors: Constanze Bonifer, [c.bonifer@bham.ac.uk](mailto:c.bonifer@bham.ac.uk); Jane Gilmour, j.gilmour@bham.ac.uk

**Contents:**

Supplementary Methods

List of Supplementary Figures and Tables

Supplementary Figures and Figure Legends

Supplementary References

**Supplementary Methods**

***ES cell differentiation***

*In vitro* haematopoietic differentiation of the iRUNX1 ES cell line using the blast culture system was performed essentially as described previously ^1^. Prior to differentiation, the iRUNX1 cell line was grown on gelatin in the absence of feeder cells for two passages. Cells were then differentiated as embryoid bodies (EBs) in 15cm bacterial-grade dishes (Sterilin) at a concentration of 1.25 x10^6^ cells per 50 ml for 3.5 days in IMDM differentiation medium without LIF (IMDM supplemented with 15% FCS, 100 units/ml Penicillin and 100 μg/ml Streptomycin, 1 mM glutamine, 0.15 mM MTG, 0.18 mg/ml human transferrin (Roche) and 50 μg/ml Ascorbic Acid. EBs were then dispersed to single cells using TrypLE Express (Gibco, Thermo Fisher) and FLK1+ cells were purified by magnetic cell sorting using a biotin-conjugated anti-FLK1 antibody (ebioscience), anti-biotin microbeads (Miltenyi Biotec) and MACS columns (Miltenyi Biotec). Purified FLK1+ cells were cultured in gelatinized T150 flasks at 1.6 x 10^6^ cells per flask in blast culture medium (IMDM supplemented with 10% FCS, 100 units per ml penicillin and 100 μg per ml streptomycin, 1 mM glutamine, 0.45 mM MTG, 0.18 mg per ml human transferrin, 25 μg per ml ascorbic acid, 20% D4T conditioned media, 5 μg/L mVEGF (Peprotech), 10 μg/L mIL-6 (Peprotech)) for 2 days. After 2 days of blast culture the haemogenic endothelium cells were purified by MACS using the FLK1 cell surface marker as described above. Cells were plated at 3 x 10^6^ cells per T150 flask in haemogenic endothelium media (IMDM supplemented with 10 % FCS, 1% KL supernatant, 100 units/ml Penicillin and 100 μg/ml Streptomycin, 1 mM glutamine, 0.15 mM MTG, 0.18 mg/ml human transferrin, 25 μg/ml ascorbic acid, 10 μg/ml Oncostatin M (R&D Systems), 1 μg/ml basic mFGF (Peprotech)) and allowed to settle for an hour before treatment with the different conditions. The HE culture was either untreated (No Dox); induced with 0.1 μg/ml Doxycyclin (Sigma) (+Dox); treated with 0.25μM JQ1 (Tocris) only (No Dox +JQ1) or treated with 0.1 μg/ml Doxycycline and 0.25μM JQ1 (+Dox +JQ1). Cells were incubated under these conditions for 18 hours before the adherent cells were harvested for RNA, FACS or ChIP analysis, with the exception of the Med12 and LDB1 ChIPs where cells were incubated for up to 40 hours.

***RNA isolation and manual RT-PCR***

RNA was extracted from cells using TRIzol (Invitrogen) according to manufacturer’s instructions. First-strand cDNA synthesis was carried out using Superscript II (Invitrogen) and Oligo dT (Invitrogen) according to manufacturer’s instructions. RNA was DNase treated using Turbo DNase (Ambion) and a further clean-up step was performed using the RNA Nucleospin kit (Macherey-Nagel) according to manufacturer’s instructions. Real-time PCR was carried out using Applied Biosystems SYBR green master mix (Thermo Fisher) with 5 μl of diluted cDNA and 0.25 μM forward and reverse primers per 10 μl reaction on an ABI 7900HT machine. Analysis was carried out on samples measured in duplicate. Statistical analysis was performed on at least 3 independent data sets using Students t-test.

**Primers used for qRT-PCR**:

Runx1: GCAGGCAACGATGAAAACTACTC

CAAACCTGAGGTCGTTGAATCTC

Spi1 (Pu.1): CCATAGCGATCACTACTGGGATTT

TGTGAAGTGGTTCTCAGGGAAGT

Gfi1: GTGAGCCTGGAGCAACACAA

CTCTTGAAGCTCTTGCCACAGA

Sox17: CTAAGCAAGATGCTAGGCAAG

TACTTGTAGTTGGGGTGGTCC

Scl (Tal1): CAACAACAACCGGGTGAAGA

ATTCTGCTGCCGCCATCGTT

ActB: AGCACAGCTTCTTTGCAGCTC

ATCAGCGCAGCGATATCGTCAT

GAPDH: ACCTGCCAAGTATGATGACATCA

GGTCCTCAGTGTAGCCCAAGAT

***Chromatin Immunoprecipitation (ChIP)***

ChIP was performed as described previously ^2-4^. Cells were harvested and washed with PBS before a 2-step crosslinking procedure. First, proteins were crosslinked by incubating cells for 45min at RT in PBS supplemented with 0.83 mg/ml Di(N-succinimidyl) glutarate (DSG, Sigma). Cells were then washed 4 times with PBS, prior to formaldehyde crosslinking of proteins and DNA for 10min at RT using 1% formaldehyde (Pierce) in IMDM with 10% FCS. Formaldehyde was quenched by adding 1/10^th^ volume 2M glycine and crosslinked cells were washed twice in ice-cold PBS. For samples to be used for histone modification ChIP, single crosslinking with 1% formaldehyde was performed. Nuclei were prepared essentially as described in Lefevre *et al* 2003, sonicated using a Bioruptor water bath in immunoprecipitation buffer I (25 mM Tris 1 M pH 8.0, 150 mM NaCl, 2 mM EDTA pH 8.0, 1% TritonX-100 and 0.25% SDS) ^5^. After centrifugation the sheared 0.5-2 kb chromatin fragments (1-2 x 10^6^ cells) were diluted with 2 volumes immunoprecipitation buffer II (25 mM Tris pH 8.0, 150mM NaCl, 2 mM EDTA pH 8.0, 1% TritonX-100, 7.5% glycerol). Immunoprecipitation was carried out for 2-4 hours at 4°C using 2 μg antibody coupled to 15 μl Protein-G dynabeads (Invitrogen), with the exception of the H3K79me2 and the H4K5Ac antibodies where immunoprecipitation was carried out overnight. Following IP, the beads were washed with low salt, high salt, LiCl and TE/NaCl buffers before crosslinks were reversed overnight. DNA was extracted using Ampure beads (Beckman Coulter) and qPCRs were performed to validate ChIP quality. Manual ChIP qPCRs were performed in duplicate on at least 3 independent sample sets.

The Pol II ChIP-seq library for the uninduced sample was previously published in Lichtinger *et al* 2012, and the Pol II +Dox library was prepared using the same method ^1^. The remaining ChIP-seq libraries were prepared from single samples using the Kapa Hyper-Prep Kit (Kapa Biosystems) according to the manufacturer’s guidelines. Libraries were size selected on a 2% agarose gel for 200-450 bp fragments, purified and re-validated by qPCR prior to sequencing. Libraries were sequenced in a pool of 12 indexed libraries using a NextSeq® 500/550 High Output Kit v2 (75 cycles) (Illumina, FC-404-2005) at the Genomics Birmingham sequencing facility.

**Antibodies used for ChIP and ChIP-seq:**

Pol II, Abcam ab817; HA (for RUNX1), Sigma H6908; LDB1, Abcam ab96799; Med12, Bethyl Labs A300-774A; BRD4, Bethyl Labs A301-985A-100; FLI1, Santa Cruz sc356; H3K79me2, Abcam ab3594; H4K5Ac, Millipore 07-327; CDK9, Santa Cruz sc8338X.

**Primers used for manual ChIP:**

Chr2: AGGGATGCCCATGCAGTCT

CCTGTCATCAGTCCATTCTCCAT

Gfi1 -35kb enhancer: CCACATGACCTCATGAATGC

CCACAAACAGAACAGCTGGA

NFE2 -3kb element: TGTTTGGCAACAATGCTTGTG

CAACCCACCTCCACTACGTAT

Oct4 promoter: TGGGCTGAAATACTGGGTTC

TTGAATGTTCGTGTGCCAAT

Tln2 intragenic: GGCAGAGACTCATTGCTCACT

CCTGTTCTGTGTCAGCGTTC

***Data Analysis***

**ChIP-seq data analysis**

Raw sequencing reads were aligned to the mouse genome version mm10 with Bowtie version 2.3.1 ^6^. The quality control statistics for all samples were obtained using FastQC software. Reads that mapped uniquely to the genome were retained and duplicated reads were removed using the MarkDuplicates function in Picard tools (http://broadinstitute.github.io/picard/). Bedtools (http://bedtools.readthedocs.org/en/latest/) were used to generate tag density profiles and were displayed using the UCSC Genome Browser ^7^.

Peaks were identified with MACS version 1.4.2 ^8^ and DFilter software ^9^ with recommended parameters (-bs=100 -ks=50 –refine). Peaks common to both peak calling methods were considered for further analysis. Peaks were annotated to their closest genes and to the gene promoter if it was within 1kb of the gene transcription start site (TSS), and as exonic, intronic or intergenic otherwise. Overlaps between ChIP-seq peaks were defined by requiring the summits of two peaks to lie within +/-200 bp.

*De novo* motif analysis was performed on peaks using HOMER ^10^. Motif lengths of 6, 8, 10, and 12 bp were identified within ± 200 bp from the peak summit. The top enriched motifs with a significant p-value score were recorded.

Clustering of ChIP-Seq datasets was carried out by first creating a union of all peaks. The number of tags that mapped to these peaks was counted in a 400bp window centred on the peak summit, and subsequently normalized to total read count. Pearson correlations were then calculated for each pair of samples using the log2 of the read counts, and hierarchically clustered with Euclidean distance using complete linkage of the correlation matrix in R.

The tag density and average profiles were generated by calculating the tag density normalised as coverage per million of the ChIP peak summit. Peaks were then ranked in order of fold-difference of the normalized tag count and plotted as a heatmap with Java TreeView (http://jtreeview.sourceforge.net/) and average profiles were plotted using R (<https://www.r-project.org/>). Each row in a heatmap represents one peak.

**RNA-seq data analysis**

RNA-Seq reads were aligned to the mouse genome version mm10 with STAR version 2.5.2b ^11^. RefSeq transcript abundances were calculated as Fragments Per Kilobase of transcript per Million mapped reads (FPKM) using Cufflinks version 2.2.1 ^12^. A gene was considered expressed if it had an FPKM value greater than 1 in at least one sample. Differentially expressed genes were generated using Cuffdiff function and genes that had a fold change greater than or equal to 2 between +Dox and no Dox or between No Dox plus JQ1 and No Dox or between +Dox+JQ1 and +Dox were considered to be differentially expressed.

Hierarchical clustering of RNA-Seq samples was carried out by first calculating the Pearson correlation coefficient of the log2 FPKM values for each pair of samples, followed by average linkage hierarchical clustering of the resulting correlation matrix in R. Correlation coefficients between replicates for the RNAseq data were above 0.98.

Clustering of differentially expressed genes was carried out on log2 FPKM values using average linkage hierarchical clustering of the Euclidean distances in R. Initially, differentially expressed genes were generated with a fold change greater than or equal to 2 between +Dox and No Dox or between No Dox plus JQ1 and No Dox or between +Dox+JQ1 and +Dox. The 3 different sets of differentially expressed genes were pooled together giving a total of 2,214 differentially expressed genes.

Gene expression fold changes were grouped according to patterns of expression between +Dox and no Dox or/and between +Dox+JQ1 and +Dox. We identified 8 groups of expression patterns, the codes of the 8 changing patterns were displayed as a heatmap where 1 denotes up-regulated, 0 denotes down-regulated and 2 denotes genes that are invariant and whose expression was unchanged. The two digit Group number refers to changes in gene expression between +Dox compared to No Dox for the first digit and changes between +Dox+JQ1 and +Dox for the second digit. For example, Group 10 genes are up-regulated in the +Dox sample compared to No Dox and down-regulated in the +Dox +JQ1 sample compared to +Dox.

Gene ontology (GO) and KEGG pathway enrichment analysis was performed using clueGO tools^13^ and the David online tool^14^ using Hypergeometric for overrepresentation and Benjamini and Hochberg (FDR) correction for multiple testing corrections. KEGG Pathway analysis using clueGO tools^13^ was performed with kappa score = 0.3. The right-sided enrichment (depletion) test based on the hypergeometric distribution was used to define GO terms.

**List of Supplementary Figures and Tables**

**Figures:**

**Supplementary Figure S1. Establishing the minimal requirement of Doxycycline in the inducible RUNX1 ES cell system.**

**Supplementary Figure S2. Deregulation of RUNX1-mediated transcription by the bromodomain inhibitor JQ1.**

**Supplementary Figure S3. Inhibition of RUNX1 binding by JQ1 selectively targets sites containing ETS motifs.**

**Supplementary Figure S4. Redistribution of FLI1 binding by RUNX1 is coupled with increased recruitment of CDK9.**

**Supplementary Figure S5. Supplementary Figure 5. RUNX1 recruits MED12 at selected genomic loci.**

**Tables:**

**Supplementary_Table_S1.xls Differentially expressed genes and GO terms.**

**Supplementary_Table_S2.xls Gene lists and GO terms for expression clusters.**

**Supplementary_Table_S3.xls Gene lists and GO terms for RUNX1 differentially expressed targets.**

**Supplementary_Table_S4.xls Group 1-5 differentially expressed genes and GO terms.**

**Supplementary_Table_S5.xls RUNX1 and FLI1 targets related to Figure 5C.**

**Supplementary Figures and Figure Legends**

**
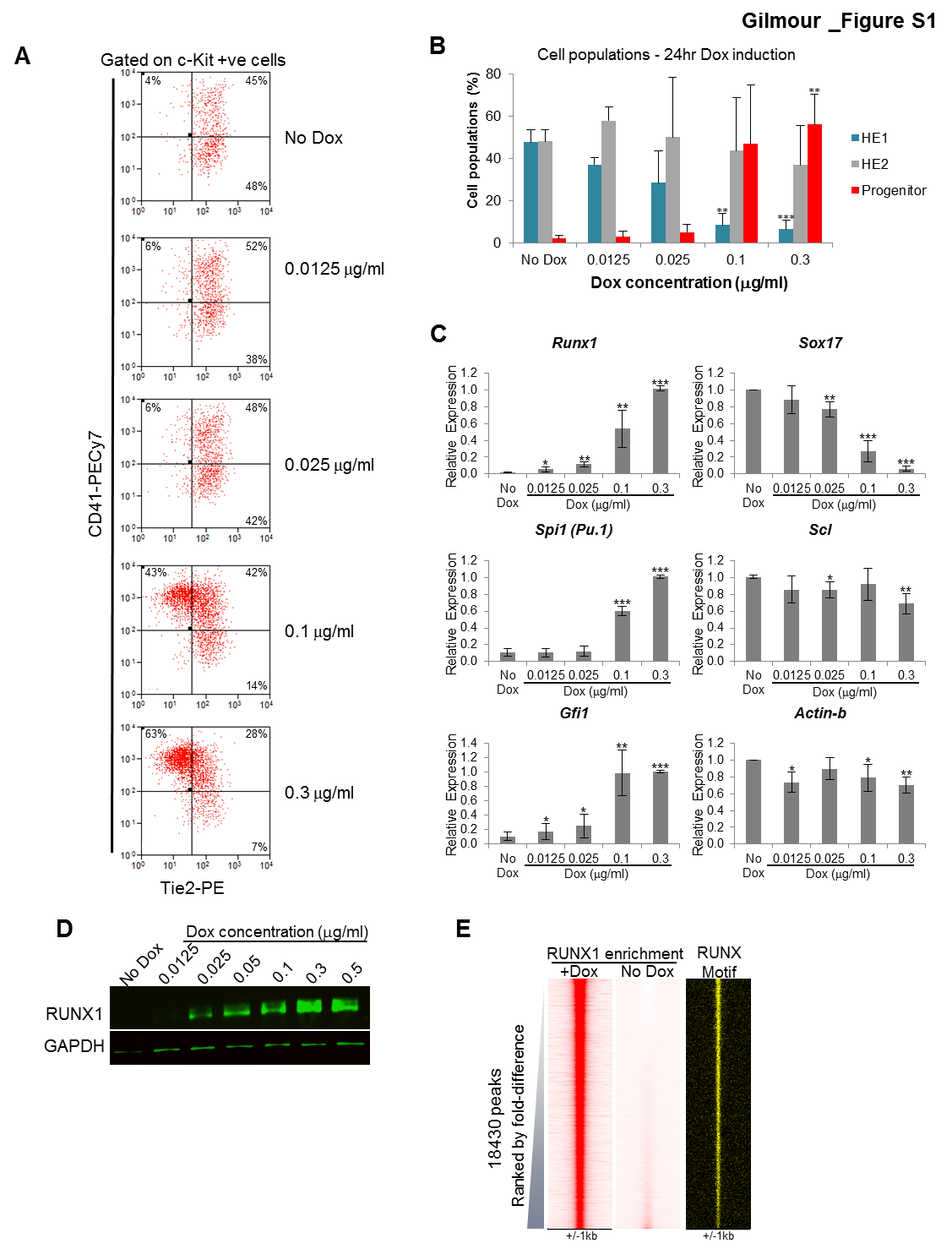
**

**Supplementary Figure S1. Establishing the minimal requirement of Doxycycline in the inducible RUNX1 ES cell system.**

A. Representative flow cytometry analysis of iRUNX1 HE treated with increasing concentrations of Dox for 24hrs. Cells were stained with c-Kit- (CD117) APC, Tie2-PE and CD41-PECy7. Cells were gated on c-Kit positive cells and Tie2 and CD41 staining within these populations is shown.

B. The proportions of HE1, HE2 and progenitor cells were calculated from FACS plots shown in Supplemental Figure S1A, based on the expression of c-Kit, CD41 and Tie2. Error bars represent standard deviation, n=3. ** denotes p<0.01, *** denotes p<0.001 when compared with the No Dox sample for each cell type using t-test.

C. Gene expression titration curve for selected genes. Normalised to *GAPDH* and either the No Dox sample or the 0.3μg/ml Dox sample. For *Runx1*, *Gfi1* and *Spi1* the calibrator used was the 0.3μg/ml Dox sample. For *Sox17*, *Scl* and *Actin-b*, the calibrator used was the No Dox sample. Error bars represent standard deviation, n=4. T-test was performed to compare the No Dox sample to each of the Dox treated samples: * represents p<0.05; ** represents p<0.01; *** represents p<0.001.

D. Western Blot showing induction of RUNX1 in HE using the indicated concentrations of Dox.

E. Heat maps showing enrichment of RUNX1 ChIP-seq binding ranked by fold difference between +Dox and No Dox samples. RUNX1 ChIP was performed using an HA antibody directed to the Dox inducible HA-RUNX1 construct. RUNX motif enrichment is shown alongside, ranked along the same co-ordinates.


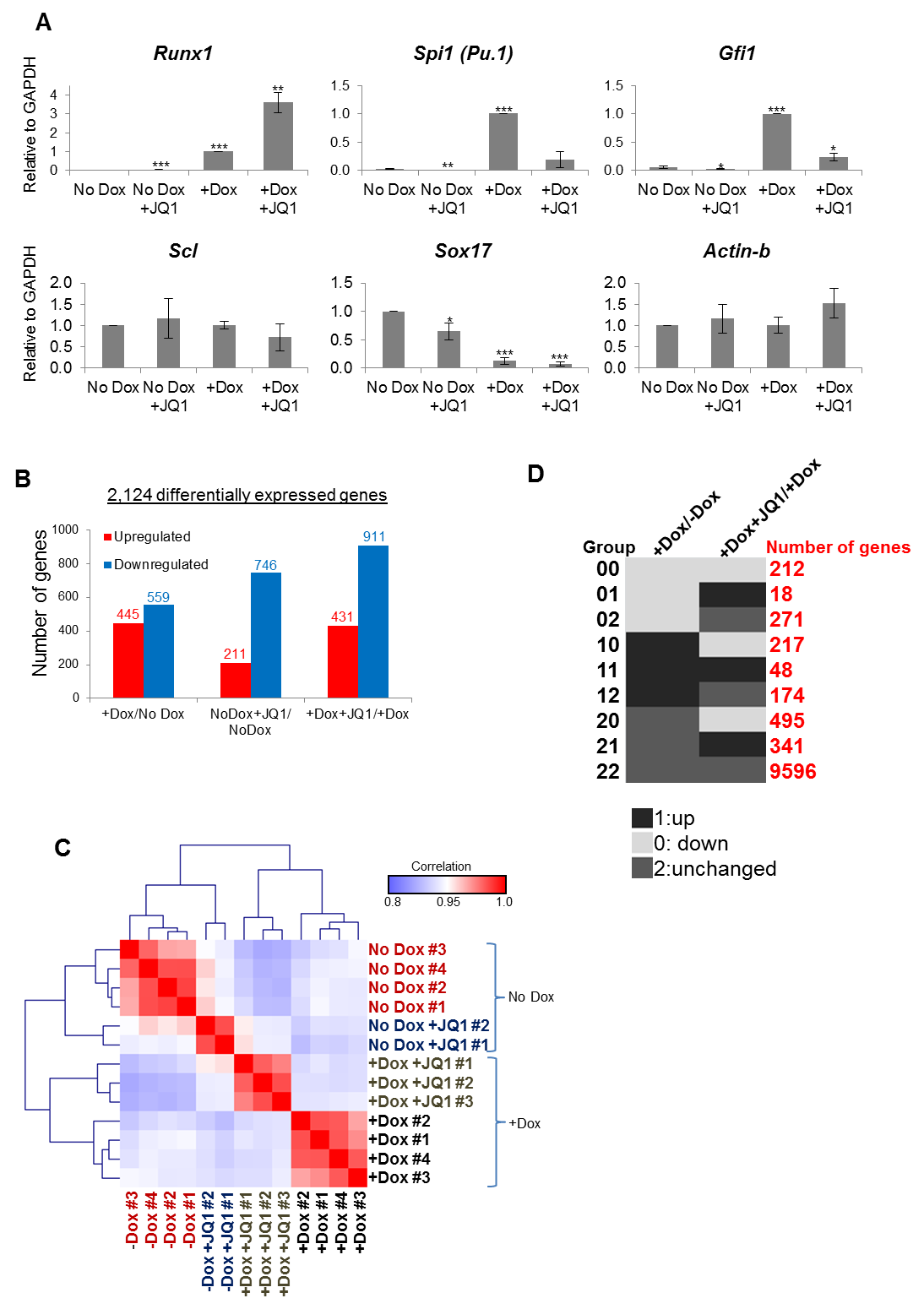


**Supplementary Figure S2. Deregulation of RUNX1-mediated transcription by the bromodomain inhibitor JQ1.**

A. Analysis of gene expression changes at individual genes under each of the indicated treatment conditions measured by qRT-PCR. Normalised to *GAPDH* and either the No Dox sample or the +Dox sample. For *Runx1*, *Gfi1* and *Spi1* the calibrator used was the +Dox sample. For *Sox17*, *Scl* and *Actin-b*, the calibrator used was the No Dox sample. Error bars represent standard deviation, n=3. * denotes p<0.05, ** denotes p<0.01, *** denotes p<0.001 when compared to the No Dox sample using Students t-test.

B. Graph showing the number of up and downregulated genes between the indicated treatment conditions: +Dox compared to No Dox; No Dox +JQ1 compared to No Dox and +Dox +JQ1 compared to +Dox. Across all three combinations 2,124 genes were found to change expression more than 2 fold. The number of genes changing expression between each treatment condition is indicated above the relevant bar. Upregulated genes are shown in red, downregulated genes are shown in blue.

C. Hierarchical clustering of the Pearson correlation coefficient of the replicates from the 4 treatment conditions. Four replicates were used for the No Dox and +Dox samples, three replicates for the +Dox +JQ1 sample and two replicates for the No Dox +JQ1 sample.

D. Grouping analysis of gene expression data as shown in Figure 2C. Data was sorted into 8 groups based on whether the gene expression was upregulated (1) or downregulated (0) more than 2 fold or unchanged (2) between the No Dox sample and the +Dox sample (left column) and between the +Dox +JQ1 sample and the +Dox sample (right column). The two digit Group number represents these changes in expression ie: 0, 1 or 2 for each column. The number of genes within each group is indicated in red in the right hand column.


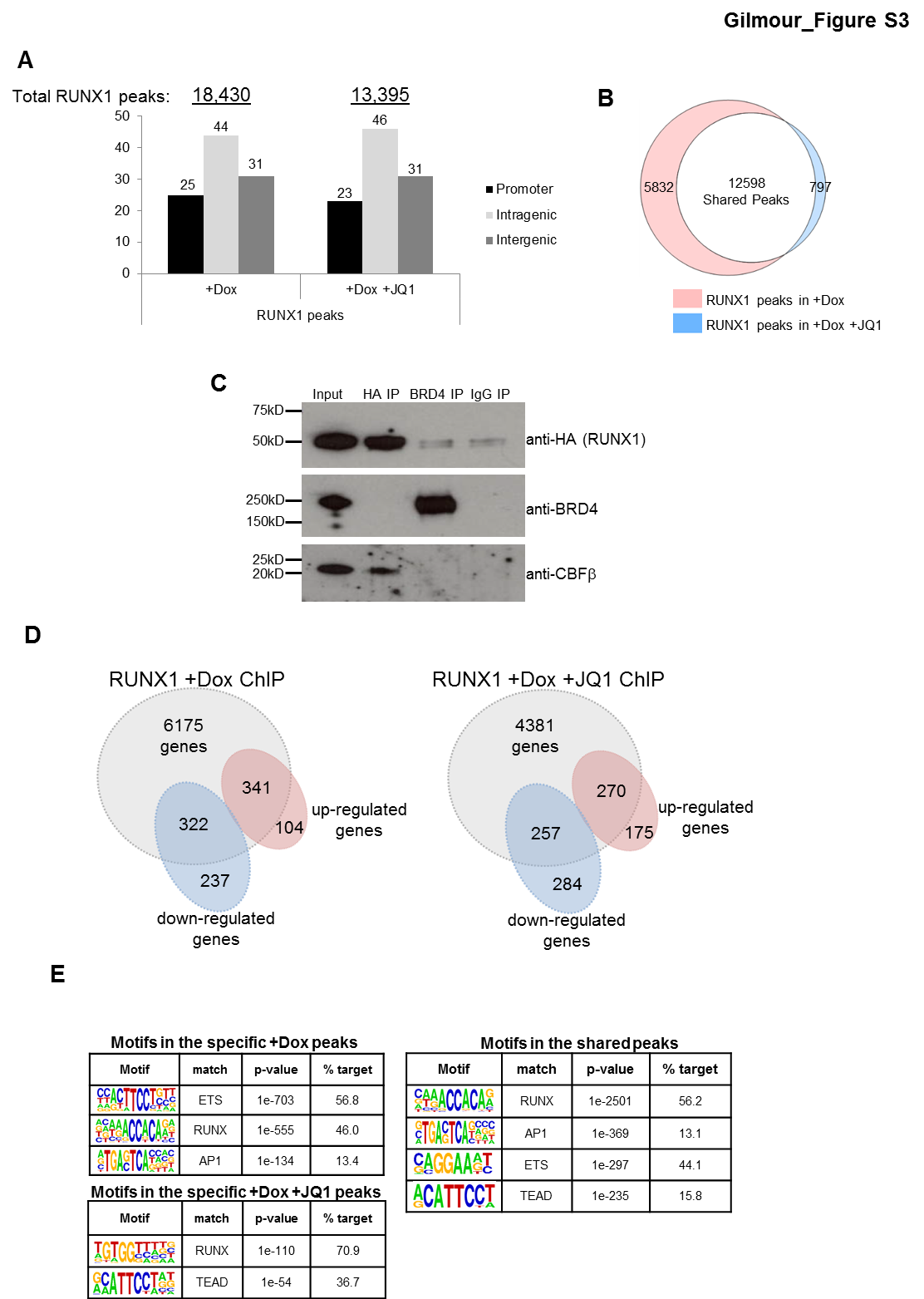


**Supplementary Figure S3. Inhibition of RUNX1 binding by JQ1 selectively targets sites containing ETS motifs.**

A. Genomic distribution of RUNX1 ChIP-seq peaks in the +Dox and +Dox +JQ1 samples. Percentage peaks are shown for promoter, intragenic and intergenic peaks. Total numbers of RUNX1 ChIP-seq peaks for each sample are shown underlined above the graph. The promoter was defined as the region between -1kb and +0.5kb. These groups are also represented in the heatmaps shown in Fig3A and 3E.

B. Venn diagram showing overlap of RUNX1 peaks in the +Dox and +Dox+JQ1 samples. Numbers of peaks are indicated for the shared and specific groups.

C. Western Blot showing co-immunoprecipitation with HA and BRD4 antibodies and an IgG control from nuclear extracts prepared from the +Dox sample. Immunoprecipitated material was immunoblotted with the indicated antibodies.

D. Left panel shows the overlap between RUNX1 target genes in +Dox cells and the up and downregulated genes in +Dox compared to No Dox samples. Right panel shows the overlap between RUNX1 target genes in +Dox +JQ1 cells and the up and downregulated genes in the +Dox +JQ1 sample compared to the +Dox sample.

E. *De novo* motif discovery using HOMER for the shared and specific peaks from RUNX1 ChIP-seq for the +Dox and the +Dox +JQ1 samples.


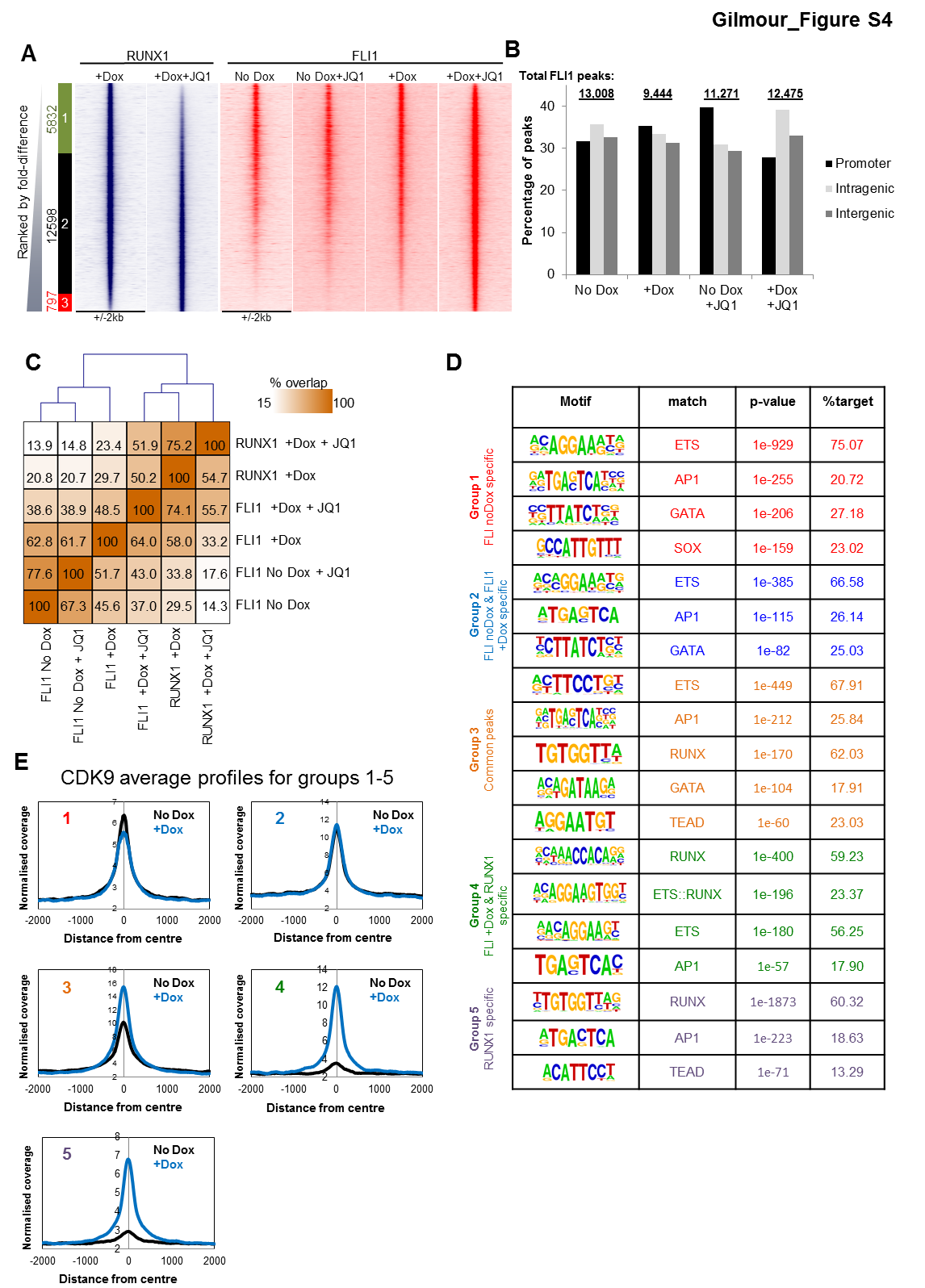


**Supplementary Figure S4. Redistribution of FLI1 binding by RUNX1 is coupled with increased recruitment of CDK9.**

A. Heat maps showing enrichment of RUNX1 and FLI1 at RUNX1 bound peaks. Ranked according to fold change of RUNX1 enrichment between the +Dox and the +Dox +JQ1 sample. Peaks and groups are defined as in Fig. 3A for RUNX1 binding, and FLI1 binding at these co-ordinates is shown alongside for each of the 4 treatment conditions.

B. Distribution of FLI1 binding between promoter, intragenic and intergenic regions in the four treatment conditions. The total number of FLI1 peaks is shown underlined above each condition.

C. Heatmap showing percentage overlap between the FLI1 and RUNX1 binding sites for all peaks.

D. *De novo* motif discovery using HOMER performed on Groups 1-5 from the overlap of FLI1 and RUNX1 distal binding sites shown in Figure 4D.

E. Average profiles of CDK9 ChIP-seq enrichment relating to Groups 1-5 for Figure 4D and Supplemental Figure S4D.


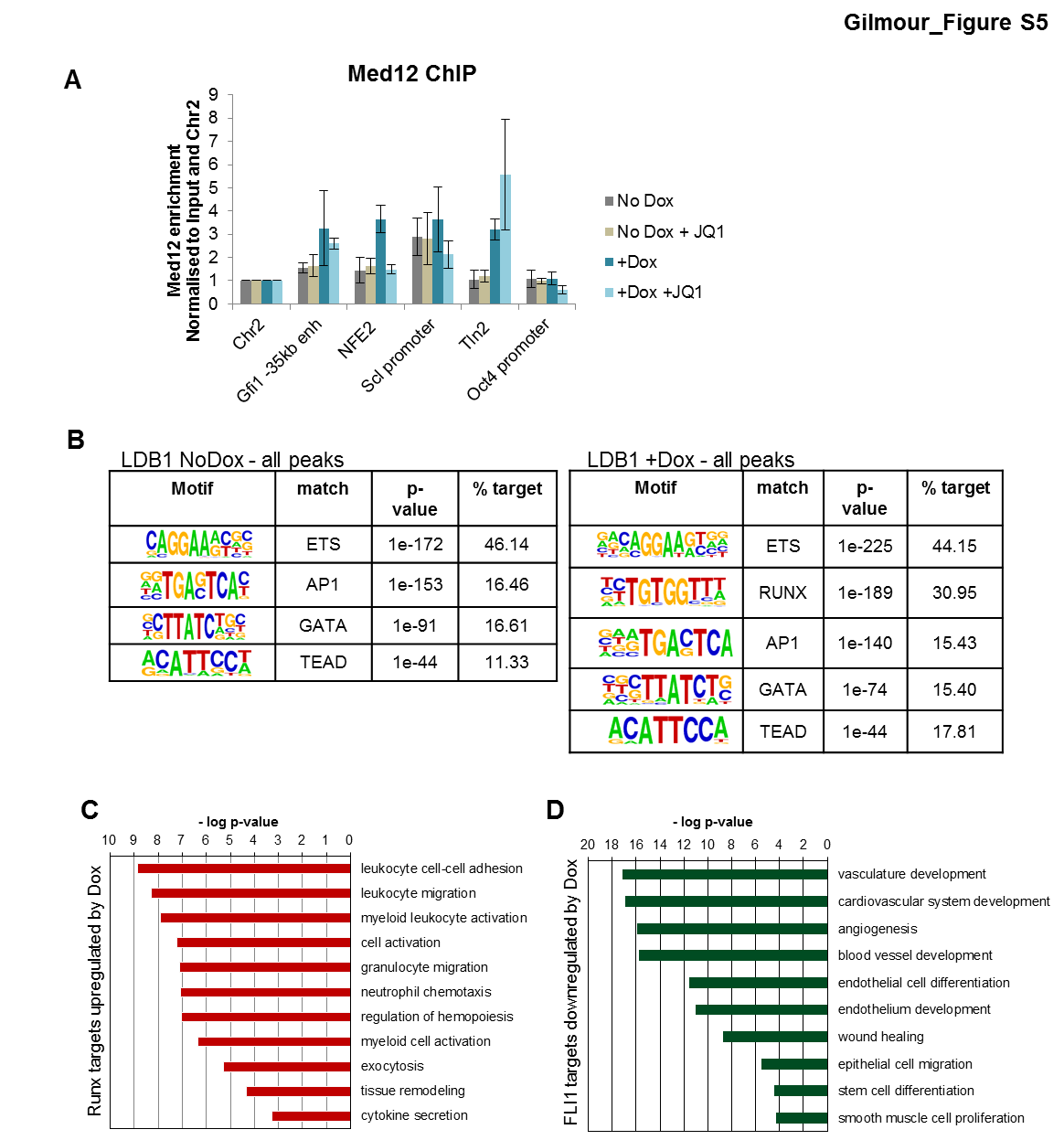


**Supplementary Figure S5. RUNX1 recruits MED12 at selected genomic loci.**

A. Manual ChIP of Med12. Error bars represent standard deviation, n=3.

B. *De novo* motif discovery using HOMER performed on LDB1 ChIP-seq peaks from the No Dox and +Dox samples shown in Figure 5B.

C. Gene ontology terms relating to Figure 5C, RUNX1 targets upregulated following Dox treatment.

D. Gene ontology terms relating to Figure 5C, FLI1 targets downregulated following Dox treatment.

**Supplementary References**

1 Lichtinger, M. *et al.* RUNX1 reshapes the epigenetic landscape at the onset of haematopoiesis. *EMBO J* **31**, 4318-4333, doi:10.1038/emboj.2012.275 (2012).

2 Gilmour, J. *et al.* A crucial role for the ubiquitously expressed transcription factor Sp1 at early stages of hematopoietic specification. *Development* **141**, 2391-2401, doi:10.1242/dev.106054 (2014).

3 Obier, N. *et al.* Cooperative binding of AP-1 and TEAD4 modulates the balance between vascular smooth muscle and hemogenic cell fate. *Development* **143**, 4324-4340, doi:10.1242/dev.139857 (2016).

4 Regha, K. *et al.* Developmental-stage-dependent transcriptional response to leukaemic oncogene expression. *Nat Commun* **6**, 7203, doi:10.1038/ncomms8203 (2015).

5 Lefevre, P., Melnik, S., Wilson, N., Riggs, A. D. & Bonifer, C. Developmentally regulated recruitment of transcription factors and chromatin modification activities to chicken lysozyme cis-regulatory elements in vivo. *Mol Cell Biol* **23**, 4386-4400 (2003).

6 Langmead, B. & Salzberg, S. L. Fast gapped-read alignment with Bowtie 2. *Nat Methods* **9**, 357-359, doi:10.1038/nmeth.1923 (2012).

7 Kent, W. J. *et al.* The human genome browser at UCSC. *Genome Res* **12**, 996-1006, doi:10.1101/gr.229102. Article published online before print in May 2002 (2002).

8 Zhang, Y. *et al.* Model-based analysis of ChIP-Seq (MACS). *Genome Biol* **9**, R137, doi:10.1186/gb-2008-9-9-r137 (2008).

9 Kumar, V. *et al.* Uniform, optimal signal processing of mapped deep-sequencing data. *Nat Biotechnol* **31**, 615-622, doi:10.1038/nbt.2596 (2013).

10 Heinz, S. *et al.* Simple combinations of lineage-determining transcription factors prime cis-regulatory elements required for macrophage and B cell identities. *Mol Cell* **38**, 576-589, doi:10.1016/j.molcel.2010.05.004 (2010).

11 Dobin, A. *et al.* STAR: ultrafast universal RNA-seq aligner. *Bioinformatics* **29**, 15-21, doi:10.1093/bioinformatics/bts635 (2013).

12 Trapnell, C. *et al.* Differential analysis of gene regulation at transcript resolution with RNA-seq. *Nat Biotechnol* **31**, 46-53, doi:10.1038/nbt.2450 (2013).

13 Bindea, G. *et al.* ClueGO: a Cytoscape plug-in to decipher functionally grouped gene ontology and pathway annotation networks. *Bioinformatics* **25**, 1091-1093, doi:10.1093/bioinformatics/btp101 (2009).

14 Huang da, W., Sherman, B. T. & Lempicki, R. A. Systematic and integrative analysis of large gene lists using DAVID bioinformatics resources. *Nat Protoc* **4**, 44-57, doi:10.1038/nprot.2008.211 (2009).
