# Supplementary material for: The Co-operation of RUNX1 with LDB1, CDK9 and BRD4 Drives Transcription Factor Complex Relocation During Haematopoietic Specification
Source: Sci Rep. 2018 Jul 10;8:10410. doi: 10.1038/s41598-018-28506-7 (PMC6039467; doi:10.1038/s41598-018-28506-7)

The Co-operation of RUNX1 with LDB1, CDK9 and BRD4 Drives Transcription Factor Complex Relocation During Haematopoietic Specification.

Jane Gilmour*, Salam A. Assi, Laura Noailles, Monika Lichtinger, Nadine Obier, and Constanze Bonifer*.

Institute of Cancer and Genomic Sciences, College of Medical and Dental Sciences, University of Birmingham, Birmingham, B15 2TT, UK.
*Corresponding Authors: Constanze Bonifer, c.bonifer@bham.ac.uk; Jane Gilmour, j.gilmour@bham.ac.uk

Relating to Supplementary Figure 1D: RUNX and GAPDH western blots

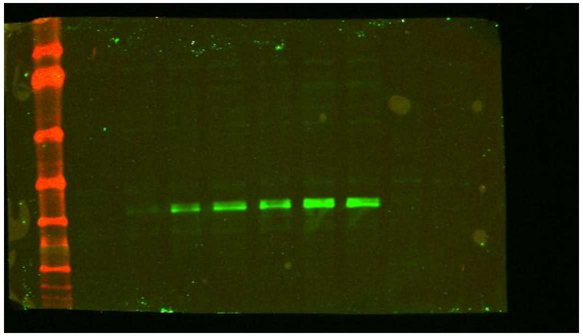

HA antibody to HA-RUNX1

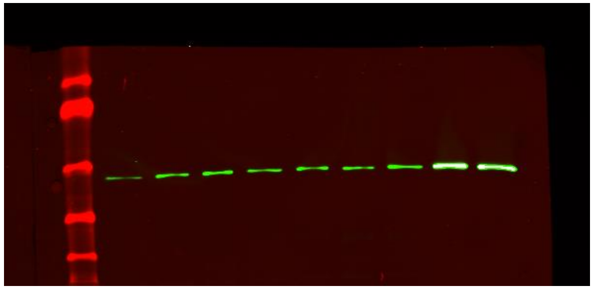

GAPDH antibody

Relating to Supplementary Figure 3C: Co-IP blots

Anti- HA (RUNX1)

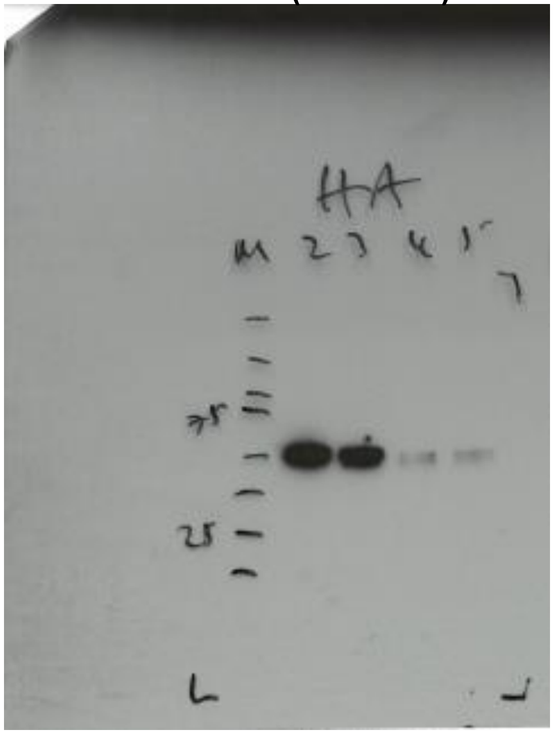

Anti- BRD4

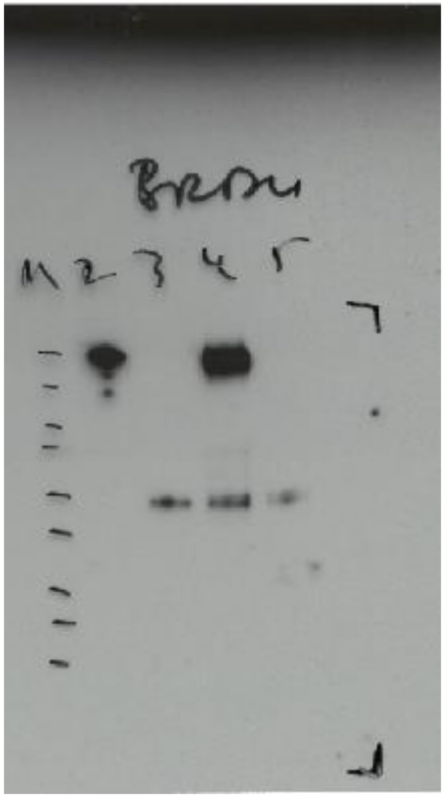

Anti- CBFβ

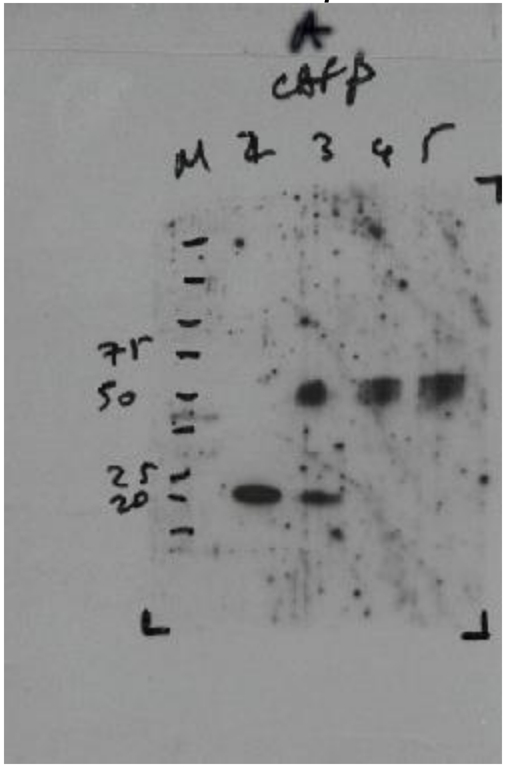

Supplement: Supplementary file 7 — Original blots [file 41598_2018_28506_MOESM7_ESM.pdf]
